# Reduced Energy Metabolism Impairs T Cell-Dependent B Cell Responses in Patients With Advanced HBV-Related Cirrhosis

**DOI:** 10.3389/fimmu.2021.660312

**Published:** 2021-06-23

**Authors:** Chunhong Huang, Junwei Shao, Congcong Lou, Fengtian Wu, Tiantian Ge, Hainv Gao, Xiaoping Zheng, Xuejun Dong, Lichen Xu, Zhi Chen

**Affiliations:** ^1^ State Key Laboratory for Diagnosis and Treatment of Infectious Diseases, National Clinical Research Center for Infectious Diseases, Collaborative Innovation Center for Diagnosis and Treatment of Infectious Disease, The First Affiliated Hospital, Zhejiang University School of Medicine, Hangzhou, China; ^2^ Department of Infectious Diseases, Shulan (Hangzhou) Hospital Affiliated to Zhejiang Shuren University Shulan International Medical College, Hangzhou, China; ^3^ Department of Pathology, Shulan (Hangzhou) Hospital Affiliated to Zhejiang Shuren University Shulan International Medical College, Hangzhou, China; ^4^ Department of Clinical Laboratory Center, Shaoxing People’s Hospital (Shaoxing Hospital, Zhejiang University School of Medicine), Shaoxing, China; ^5^ Department of Nephrology, Sir Run Run Shaw Hospital, Zhejiang University School of Medicine, Hangzhou, China

**Keywords:** cirrhosis, B cell, metabolism, OCR, ECAR, mTOR

## Abstract

**Background and Aims:**

Patients with decompensated HBV-related liver cirrhosis (HBV D-LC) showed compromised immune responses, which manifested as a proneness to develop infections and hyporesponsiveness to vaccines, resulting in accelerated disease progression. The alterations in T cell-dependent B cell responses in this pathophysiological process were not well understood. This study aimed to investigate T cell-dependent B cell responses in this process and discuss the mechanism from the perspective of metabolism.

**Methods:**

Changes in phenotypes and subsets of peripheral B cells between HBV D-LC patients and healthy controls (HCs) were compared by flow cytometry. Isolated B cells were activated by coculture with circulating T follicular (cTfh) cells. After coculture, the frequencies of plasmablasts and plasma cells and immunoglobin levels were analyzed. Oxidative phosphorylation (OXPHOS) and glycolysis were analyzed by a Seahorse analyzer. Mitochondrial function and the AKT/mTOR pathway were analyzed by flow cytometry.

**Results:**

The proliferation and differentiation capacities of B cells after T cell stimulation were impaired in D-LC. Furthermore, we found that B cells from D-LC patients showed reductions in OXPHOS and glycolysis after activation, which may result from reduced glucose uptake, mitochondrial dysfunction and attenuated activation of the AKT/mTOR pathway.

**Conclusions:**

B cells from HBV D-LC patients showed dysfunctional energy metabolism after T cell-dependent activation. Understanding the regulations of B cell metabolic pathway and their changes may provide a new direction to rescue B cell hyporesponsiveness in patients with HBV D-LC, preventing these patients be infected and improving sensitivity to vaccines.

## Highlights

- B cell proliferation and differentiation were attenuated after T cell-dependent activation in HBV-related cirrhosis.
- Metabolic impairment results in B cell dysfunction after activation.
- Reduced activity of the AKT/mTOR pathway led to B cell metabolic impairment.

## Introduction

Cirrhosis is the end stage of all liver diseases and is the 14th most common cause of death worldwide (1). Cirrhotic patients are susceptible to bacterial infections, which may lead to acute decompensation (AD) and acute-on-chronic liver failure (ACLF), both of which are associated with high short-term mortality (2, 3). In recent decades, cirrhosis has been viewed as a multisystemic disease. Local and systemic inflammatory changes in the body are closely linked to the progression of disease. The immune dysfunction associated with cirrhosis starts with the onset of chronic liver inflammation, becomes worse with the development of portal hypertension, further deteriorates due to gut bacterial translocation, and ultimately culminates in immune paralysis in ACLF. However, the mechanisms of impaired immunity in cirrhotic patients are complicated and incompletely understood.

Immune dysfunction in cirrhosis involves both adaptive and innate immunity. Innate immune dysfunction has been relatively comprehensively investigated. To date, evidence has indicated that monocyte/macrophage, neutrophil, eosinophil, basophil, mastocyte, innate lymphoid cell, and mucosal-associated invariant T (MAIT) cell alterations contribute to the susceptibility of cirrhotic individuals to infection (4–6). Studies have shown that macrophages and neutrophils in cirrhotic patients have impaired antimicrobial functions, such as decreased pathogen recognition by pattern recognition receptors (PRRs) and attenuated phagocytosis, but high secretion of proinflammatory cytokines (5, 7).

Investigations on the humoral immune response in cirrhosis, which also plays vital roles in restricting pathogens, are limited. The weak T cell-dependent B cell response in cirrhosis has long been recognized. Cirrhotic patients with hepatitis A virus (HAV) vaccination display reduced seroconversion percentages and antibody titers (8). The response to HBV vaccination is reduced in patients with cirrhosis (9). A decrease in the CD27^+^ memory subgroup among cirrhotic B cells may contribute to this effect (10). Regarding B cell functional changes after stimulation, the conclusions were contradictory: one result showed that activation of peripheral B cells in cirrhosis was attenuated after CD40/TLR9 stimulation, manifesting as reductions in CD70 expression and IgG production (10), and the other result demonstrated that cirrhotic B cells were prone to differentiate into plasma cells and produce increased amounts of immunoglobulins (11). Although the damaged humoral response in cirrhosis has been confirmed, the mechanism and B cell response after activation, especially the T cell-dependent B cell response, remain to be further explored, as T cell help plays a pivotal role in humoral immunity.

It is now understood that changes in B cells from resting to an active state after stimulation require metabolic reprogramming, which provides both fuel to meet the energy requirements of highly active cells and intermediates for biosynthesis (12–14). During metabolic reprogramming, both the level of oxidative phosphorylation (OXPHOS) and glycolysis are increased, demonstrating their crucial roles in B cell activation (12, 13). The expression of glucose transporter 1 (GLUT1), a membrane glucose transporter, and glucose uptake were also increased in activated B cells (12, 15).

As cellular metabolism contributes significantly to lymphocyte development and activation (16), we hypothesized that alterations in metabolic patterns and reductions in energy supply may play pivotal roles in B cell hyporesponsiveness after activation by T cells. To test this hypothesis, we investigated the proliferation and differentiation abilities of HBV D-LC B cells in a Tfh cell helping model and further clarified the mechanisms of B cell hyporesponsiveness after T cell-dependent activation from an energy metabolism perspective.

## Materials and Methods

### Study Subjects

In total, 132 patients with HBV-related cirrhosis were enrolled in this study, including 114 decompensated liver cirrhosis (D-LC) patients and 18 compensated liver cirrhosis (C-LC) patients. Cirrhosis was diagnosed according to a liver biopsy, radiological evidence and clinical findings, as previously described (17). Cirrhotic patients who presented one or more symptoms of ascites, hepatic encephalopathy or upper gastrointestinal bleeding were defined as decompensated. The exclusion criteria included the following: (1) cirrhosis caused by other etiologies, (2) human immunodeficiency virus (HIV) infection, (3) cancer, (4) immunosuppressive therapy (cytotoxic or corticosteroid treatment), and (5) autoimmune diseases. Demographic and clinical information are summarized in **Table 1**. Healthy controls (HCs) matched by age and sex were recruited from the Physical Examination Center at the same time. All subjects were recruited from the First Affiliated Hospital, Zhejiang University School of Medicine. Written informed consent was obtained from individual subjects, and the experimental protocol was approved by the Ethics Committee of the same hospital.

**Table 1:** The demographic and clinical characteristics of cirrhosis subjects.

Variables	C-LC (n = 18)	D-LC (n = 114)
Age (y)	56 (52–64.5)	54.5 (49–3)
Male/Female	9/9	85/29
Laboratory parameters		
ALT (U/L)	19.0 (16.0–28.5)	22.0 (13.0–41.0)
AST (U/L)	27.0 (20.5–33.5)	39.0 (24.0–54.5)
TBIL (μmol/L)	12.4 (8.65–24.3)	26.9 (16.5–61.7)
INR	n.a.	1.39 (1.21–1.56)
Creatinine (μmol/L)	69.0 (60.0–77.5)	71.0 (56.0–88.5)
Decompensation events		
Ascites (%)	0(0)	78 (78.8)
Upper gastrointestinal hemorrhage (%)	0(0)	24 (24.2)
Hepatic encephalopathy (%)	0(0)	4 (4.0)
Severity score		
MELD	–	13.3 (10.3–17.0)

Data were shown as median (interquartile range) or case number.

ALT, alanine aminotransferase; AST, aspartate aminotransferase; TBIL, total bilirubin; INR, international normalized ratio; MELD, Model for End-Stage Liver Disease; n.a., not available.

### Cell Isolation

Peripheral blood mononuclear cells (PBMCs) were isolated using Ficoll density gradient centrifugation. CD19^+^ B cells, naïve B cells (CD19^+^CD27^-^) and memory B cells (CD19^+^CD27^+^) were directly isolated by magnetic microbeads separation kits. To isolate circulating T follicular (cTfh) cells, CD4^+^ T cells were negatively selected by magnetic microbeads isolation kit first, and then the separated CD4^+^ T cells were incubated with PE-labeled anti-CXCR5 antibodies and isolated with anti-PE magnetic microbeads. cTfh cells were the positive population. All isolation kits were purchased from Miltenyi Biotech and used according to the user guide. The cell purity was assessed by flow cytometry. Memory B cells and naïve B cells with purity >85% were used in the following experiments, and other cells with purity >90%. More details were shown in **Supplementary Methods** and **Supplementary Figure 1**.

### *In Vitro* T-B Cell Coculture System

Freshly isolated B cells (3 × 10^4^/well) from HC or D-LC were cocultured with allogeneic healthy cTfh cells (3 × 10^4^/well) in the presence of staphylococcal enterotoxin B (SEB) (50 ng/ml) for 6 or 9 d. In some experiments, B cells were pretreated with everolimus (1 μM), PD98059 (10 μM), pimozide (7.5 μM) or wortmannin (10 μM) for one day and then cocultured with cTfh cells after the inhibitors were removed. The cells were analyzed by flow cytometry, and the supernatants were collected for immunoglobin analysis by a precoated enzyme-linked immunosorbent assay (ELISA) kit (Multisciences).

### Flow Cytometry

For surface staining, cells were stained with fluorescent-conjugated Abs at room temperature for 15 min. For intracellular phosphorylated protein staining, cells were stained with LIVE/DEAD™ fixable dye (catalog 65-0865-14, eBioscience) at 4°C for 20 min, fixed with Cytofix™ fixation buffer (catalog 554655, BD Bioscience) at 4°C for 20 min, permeabilized with permeabilization buffer (catalog 00-5123-43, eBioscience) at 4°C for 20 min, and then stained with fluorescent-conjugated Abs at room temperature for 15 min. For Ki67 detection, cells were fixed and permeabilized with Fixation/Permeabilization Concentrate (catalog 00-5123-43, eBioscience) at 4°C for 30 min and then stained with fluorescent-conjugated Abs at room temperature for 15 min. All analyses were carried out by a Fortessa (BD Bioscience, Franklin Lakes, NJ). All Abs used are listed in **Supplementary Table 1**.

### B Cell Activation

In the B cell activation assay, freshly isolated B cells (8 × 10^4^/well) were activated for one day with anti-IgG and IgM Abs (Thermo Fisher) (5 μg/ml) plus recombinant CD40L (Peprotech) (0.2 μg/ml) or CPG-ODN 2006 (InvivoGen) (0.5 μg/ml) as a second signal.

### Seahorse Assays

Activated CD19^+^ B cells were washed twice, resuspended in XF RPMI supplemented with 1 mM pyruvate, 2 mM L-glutamine and 10 mM glucose, and plated at 1 × 10^5^ per well on extracellular flux assay plates (Agilent Technologies) precoated with 22.4 μg/ml Cell-Tak (Corning). For the mitochondrial stress analysis, the oxygen consumption rate (OCR) was measured before and after the sequential addition of 1 μM oligomycin, 2 μM FCCP and 0.5 μM Rot/AA using a Seahorse XF96 extracellular flux analyzer (Agilent Technology). For the glycolysis stress test, the extracellular acidification rate (ECAR) was measured before and after the sequential addition of 10 mM D-glucose, 1.5 μM oligomycin, and 50 mM 2-deoxyglucose.

### Mitochondrial Function Analysis and Glucose Uptake

Mitochondrial membrane potential was detected by JC-1 (Beyotime). Freshly isolated or after culture, B cells stained with JC-1 working solution at 37°C for 20 min. 2NBDG (Glpbio) (working concentration 25 μg/ml, incubating at 37°C for 30 min) was used to assess glucose uptake by B cells. MitoSOX™ Red (Thermo Fisher) (working concentration 1 μM, incubating at 37°C for 10 min) was used to measure mitochondrial superoxide. MitoTracker™ Green (Thermo Fisher) (working concentration 25 nM, incubating at 37°C for 30 min) was used to measure mitochondrial mass. After all the staining, DAPI was used to exclude the dead cells. All parameters were analyzed by flow cytometry.

### Statistical Analysis

The flow cytometry results were analyzed by FlowJo 10.7.1 (BD Bioscience) and were concatenated and analyzed using the FlowJo plugins Downsample and tSNE according to the default settings. GraphPad Prism 6.0 was used for all statistical analyses. Nonnormally distributed data are presented as the median and interquartile range and were analyzed by the Mann–Whitney U test. Normally distributed data are shown as the mean ± SD and were analyzed using Student’s t-tests. Differences were considered significant at a two-sided p value ≤0.05.

## Results

### Alterations in B Cell Subsets and Chemokine Receptor Expression in Decompensated Cirrhosis Patients

**Figure 1:**
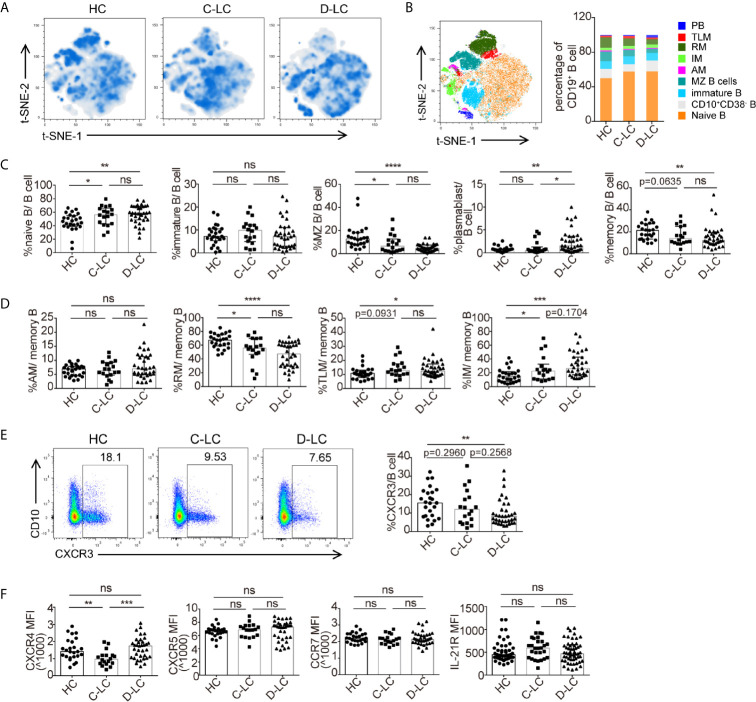
The subsets and phenotypic changes of CD19^+^ B cells in decompensated liver cirrhosis. **(A)** t-SNE projection of concatenated CD19^+^ B cells from HCs and patients with C-LC and D-LC. **(B)** Relative abundance of B cell subgroups in HCs and patients with C-LC and D-LC. **(C)** Statistical analysis of B cell subgroup changes between HCs and patients with C-LC and D-LC. **(D)** Statistical analysis of memory B cell subgroup changes between HCs and patients with C-LC and D-LC. **(E)** The representative charts and statistical analysis of CXCR3^+^ B cell frequency in CD19^+^ B cells. **(F)** Statistical analysis of MFI of CXCR4, CXCR5, CCR7 and IL-21R expression in CD19^+^ B cells in HCs and patients with C-LC and D-LC. The data are shown as the median. ns, no statistical significance. HC, n = 25; C-LC, n = 18; D-LC, n = 35. *p <0.05; **p <0.01; ***p <0.001; and ****p <0.0001.

To examine B cell phenotypes in D-LC caused by HBV, we performed flow cytometry using a panel of 11 markers (18) (CD19, CD10, CD38, IgD, CD21, CD27, CCR7, CXCR3, CXCR4, CXCR5, and IL-21R) and demonstrated the changes of B cell subsets for the first time in HBV-associated advanced cirrhosis. The gating strategy of this panel was shown in **Supplementary Figure 2**. Representative figures after t-SNE calculations are shown (**Figure 1A**). In detail, the percentages of naïve B cells and plasmablasts showed an increasing trend from HCs, C-LC patients to D-LC patients, while the percentages of marginal zone B cells and memory B cells were decreased (**Figures 1B, C** **)**. The ratio of immature B cells did not change in the context of cirrhosis (**Figures 1B, C** **)**. According to the expression of CD21 and CD27, memory B cells can be divided into active memory B cells (AMs, CD21^−^CD27^+^), resting memory B cells (RMs, CD21^+^CD27^+^), tissue-like memory B cells (TLMs, CD21^−^CD27^−^) and intermediate memory B cells (IMs, CD21^+^CD27^−^) to show the different maturation states (18). In D-LC patients, the distribution was skewed toward TLMs and IMs, with a decrease in RMs (**Figure 1D**).

As B cells should undergo chemotaxis to lymphocytic tissue to undergo further activation after encountering antigens (Ags), which could change the number and ratio of B cells and their subsets in the periphery, we also examined the chemokine receptors CXCR3, CXCR4, CXCR5, and CCR7. The percentage of CXCR3^+^ B cell was decreased in D-LC CD19^+^ B cells compared with that in HCs (**Figure 1E**). The expression of CXCR4 was decreased in C-LC patients compared with HCs and D-LC patients, while there was no difference between HCs and D-LC patients (**Figure 1F**). As for CXCR5 and CCR7, the MFI of CXCR5 and CCR7 was not different between HC, C-LC and D-LC (**Figure 1F**). The expression of the cytokine receptor of IL-21 (IL-21R), which is the main cytokine associated with B cell proliferation and differentiation, was also analyzed, and there was no difference in D-LC patients in comparison with HCs (**Figure 1F**).

### B Cells From Decompensated Cirrhosis Patients Have Attenuated Proliferation and differentiation Abilities After T Cell-Dependent Activation

**Figure 2:**
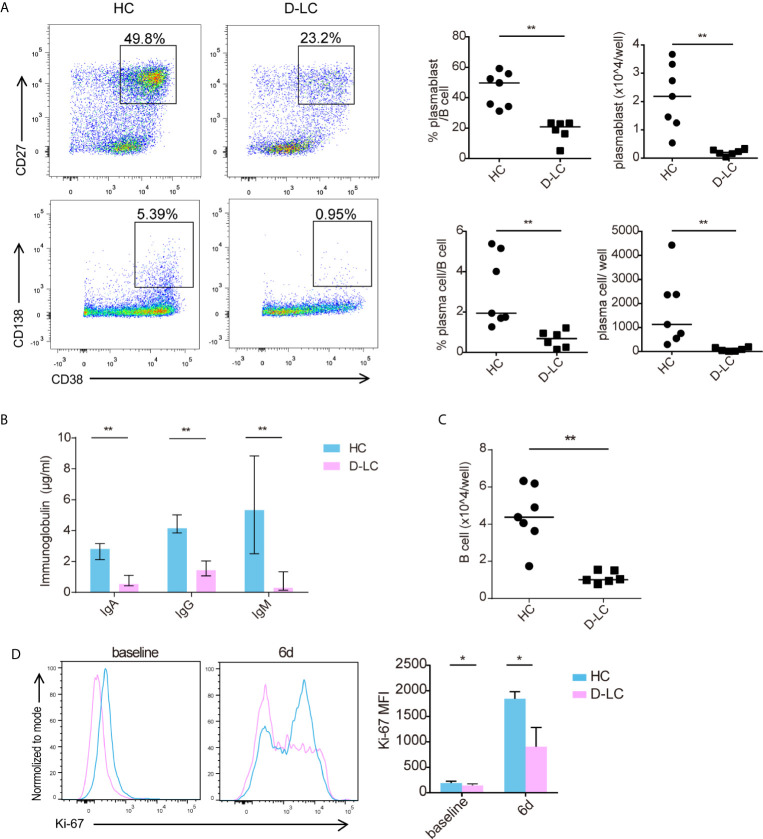
Differentiation and proliferation of CD19^+^ B cells was impaired in D-LC. Freshly isolated CD19^+^ B cells from HCs and D-LC patients were cocultured with healthy cTfh cells and treated with SEB for 6 d. **(A)** Representative flow cytometry graphs and bar charts show the percentages and numbers of plasmablasts (CD38^+^CD27^+^) and plasma cells (CD38^+^CD138^+^) among CD19^+^ B cells. **(B)** The levels of IgA, IgG and IgM in the coculture supernatants. **(C)** The number of B cells after coculture. **(D)** Ki-67 expression of CD19^+^ B cells before and after coculture. HC, n = 7; D-LC, n = 6. The data are shown as the median. *p <0.05; **p <0.01.

The specific immune response of B cells requires the help of T follicular cells and the formation of immune synapses. To mimic the help of T follicular cells, we established an *in vitro* coculture system in which B cells and cTfh cells were cultured in the presence of SEB, which acted as a superantigen to join both cell types. After 6 d of coculture, B cells from D-LC patients showed impaired differentiation compared with those from HCs, with reduced plasmablasts (CD38^+^CD27^+^) and plasma cells (CD38^+^CD138^+^) both in percentages and counts (all p <0.01) (**Figure 2A**). IgG, IgA, and IgM secreted into the supernatant was also decreased in the D-LC group compared with the HC group (all p <0.01) (**Figure 2B**). The number of total B cells after 6 d of coculture was also analyzed, and the D-LC group had a significantly smaller number of B cells than the HC group (**Figure 2C**), which suggested poor proliferation abilities of D-LC patient B cells. Ki-67, a protein that is strictly associated with cell proliferation, was further measured. D-LC B cells expressed lower levels of Ki-67 both at baseline and after 6 d of coculture (**Figure 2D**), which showed the attenuation of proliferation.

**Figure 3:**
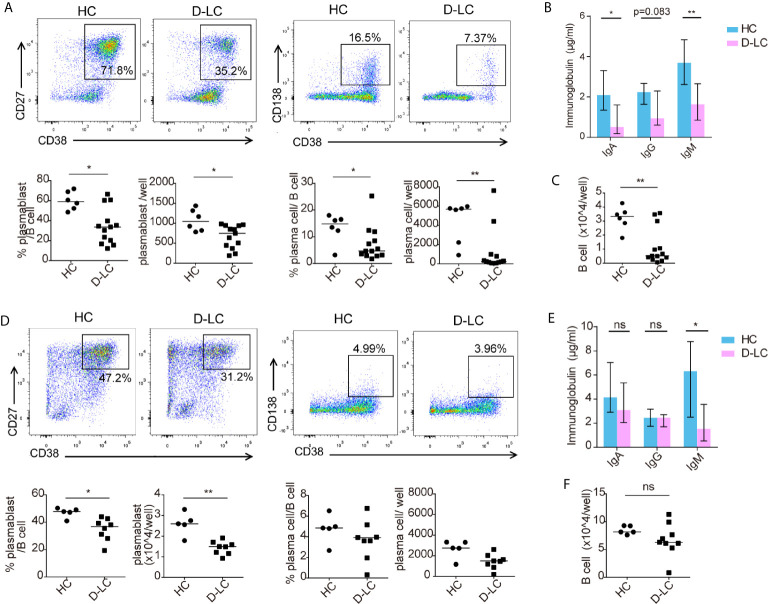
Both naïve and memory B cells showed impaired differentiation after T cell-dependent activation in D-LC. Purified naïve B cells and memory B cells from HCs and D-LC patients were cocultured with healthy cTfh cells in the presence of SEB for 9 or 6 d. Representative flow cytometry graphs and bar charts show the percentages and numbers of plasmablasts (CD38^+^CD27^+^) and plasma cells (CD38^+^CD138^+^) among naïve B cells **(A)** and memory B cells **(D)**. The levels of IgA, IgG and IgM in the coculture supernatants of naïve B cells **(B)** and memory B cells **(E)**. The numbers of naïve B cells **(C)** and memory B cells **(F)** after coculture are shown. HC, n = 6; D-LC, n = 13 for **(A–C)**. HC, n = 6; D-LC, n = 10 for **(D–F)**. The data are shown as the median. ns, no statistical significance; *p <0.05; **p <0.01.

As shown in **Figure 1B**, D-LC patients had a lower percentage of memory B cells and a higher ratio of naïve B cells than HCs. To exclude this influence, sorted memory B cells and naïve B cells from D-LC patients and HCs were cocultured separately with cTfh cells. Consistent with total B cells, naïve B cells showed significantly lower percentages and smaller numbers of both plasmablasts (CD38^+^CD27^+^) and plasma cells (CD38^+^CD138^+^) after coculture (**Figure 3A**). B cell secretion of IgG, IgA, and IgM into the supernatant was also decreased in the D-LC group in comparison with the HC group (**Figure 3B**). The total number of B cells from D-LC patients was also reduced after coculture (**Figure 3C**). For memory B cells, there was only a significant difference in the percentage and number of plasmablasts (CD38^+^CD27^+^) (**Figure 3D**) and IgM secretion (**Figure 3E**), not in the total number of B cells (**Figure 3F**), which meant that the impairments in memory B cell proliferation and differentiation in D-LC patients was not as severe as those in naïve B cells.

### Oxidative Phosphorylation and Glycolysis Were Impaired in B Cells From Decompensated Cirrhosis Patients After T Cell-Dependent Activation

**Figure 4:**
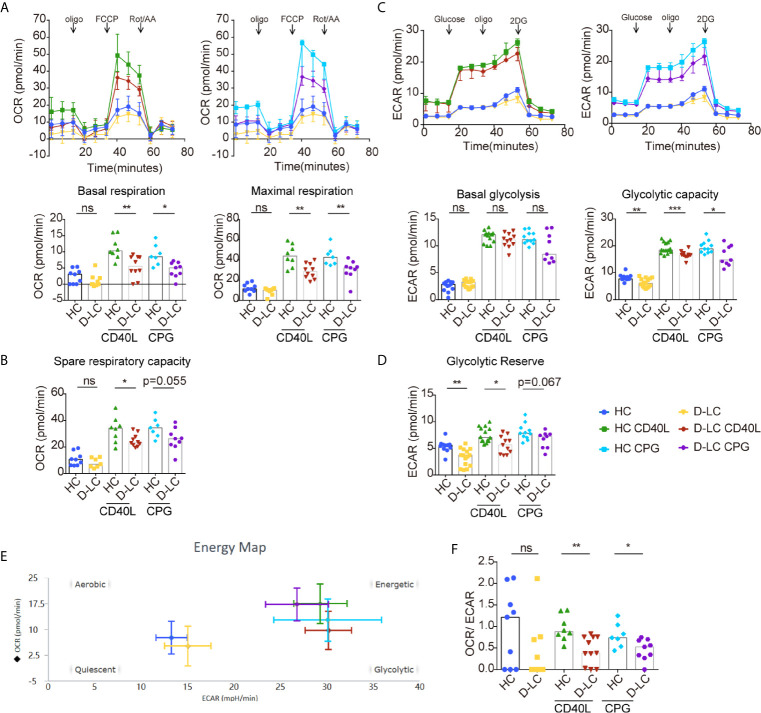
CD19^+^ B cells showed impaired oxidative phosphorylation and glycolysis after T cell-dependent activation in D-LC. Isolated CD19^+^ B cells from HCs or D-LC patients were cultured in medium alone or treated with anti-IgG,IgM Ab (5 μg/ml) and recombinant CD40L (0.2 μg/ml) or CPG-ODN 2006 (0.5 μg/ml) as a second signal for one day. Then, the OCR was assessed using a Seahorse analyzer by the sequential addition of 1 μM oligomycin, 2 μM FCCP and 0.5 μM Rot/AA (HC, n = 6; D-LC, n = 5) **(A)**. Basal respiration was determined by OCR prior to addition of oligomycin minus OCR after Rot/AA injection, and maximal respiratory capacity was determined by maximal OCR after FCCP injection subtracting OCR after Rot/AA injection. **(B)** Spare respiratory capacity, which is maximal respiratory capacity minus basal respiration, represents cellular responses to stress. For the glycolysis stress test, ECAR was assessed before and after the sequential addition of 10 mM D-glucose, 1.5 μM oligomycin, and 50 mM 2-deoxyglucose (HC, n = 8; D-LC, n = 9) **(C)**. Basal glycolysis was determined by ECAR after addition of glucose subtracting ECAR before glucose addition, and glycolytic capacity was calculated by ECAR after addition of oligomycin subtracting ECAR before glucose addition. **(D)** Glycolytic reserve was glycolytic capacity minus basal glycolysis. **(E)** The energy phenotype at baseline or after activation is shown. **(F)** Basal OCR/basal ECAR are shown. The data are shown as the median. ns, no statistical significance; *p <0.05; **p <0.01; and ***p <0.001.

B cells undergo metabolic reprogramming upon activation, with a profound increase in both oxidative phosphorylation (OXPHOS) and glycolysis (12). Isolated B cells were activated with anti-IgG, IgM, and CD40L or CPG OND 2006 was added as a second signal, as different second signals showed different effects on B cells (15). The basal and maximal respiration of unstimulated B cells was the same between HCs and D-LC patients (**Figure 4A**). After activation, the OCRs representing both basal and maximal respiration were profoundly increased, but the OCR of D-LC B cells did not increase much compared with that of HC B cells (**Figure 4A**). The spare respiration capacity, which was important for cellular responses to stress, was lower in D-LC B cells compared with HC B cells, although there was only statistical significance in CD40L treated group (**Figure 4B**). Regarding glycolysis, B cells from HCs and D-LC patients showed the same ECAR levels at both baseline and after activation (**Figure 4C**). When oligomycin was added to inhibit OXPHOS and analyze glycolytic capacity, differences were observed. B cells from D-LC patients showed obviously lower ECAR levels before and after activation than those from HCs (**Figure 4C**). The glycolytic reserve, which indicated the capability of a cell to respond to glycolysis demand, was decreased in D-LC B cells before and after activation (**Figure 4D**). This was in accordance with the phenomenon that there were no differences in basal glycolysis between HC and D-LC B cells but significant differences in glycolytic capacity. Moreover, there was no significant effect on the OCR or ECAR in response to different second signals (**Figures 4A, C** **)**.

Basal OCR and ECAR before and after activation are shown in **Figure 4E**. The ratio of basal OCR/ECAR was also analyzed and was reduced in D-LC B cells after activation (**Figure 4F**), which suggested that OXPHOS was attenuated in D-LC B cells to a large extent.

### Glucose Uptake and Mitochondrial Function Were Impaired in B Cells From Decompensated Cirrhosis Patients After T Cell-Dependent Activation

**Figure 5:**
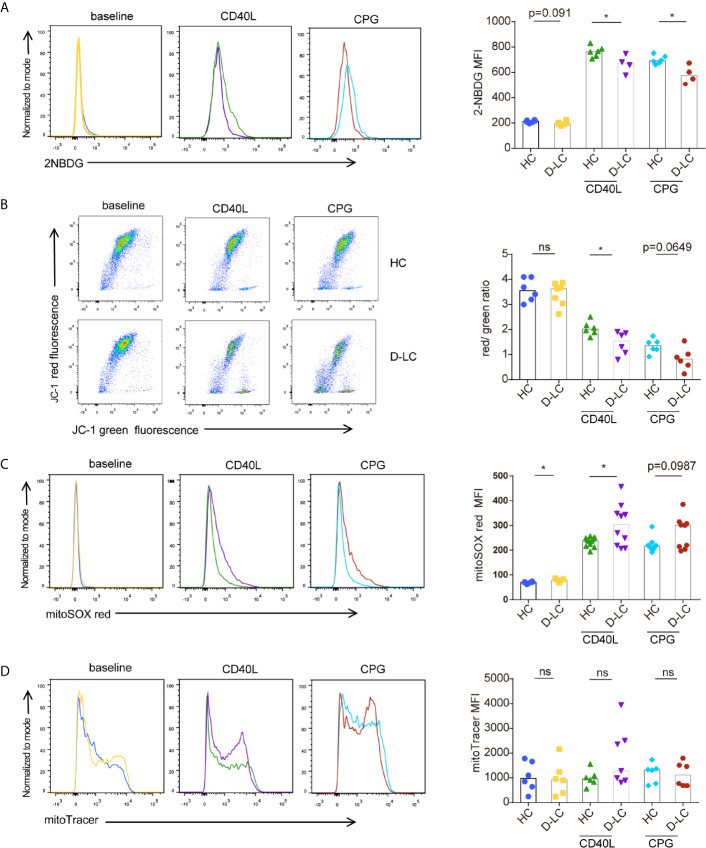
CD19^+^ B cells showed mitochondrial dysfunction at baseline and after T cell-dependent activation in D-LC. Isolated CD19^+^ B cells from HCs or D-LC patients were cultured in medium alone or treated with anti-IgG,IgM Ab (5 μg/ml) plus recombinant CD40L (0.2 μg/ml) or CPG-ODN 2006 (0.5 μg/ml) as a second signal for one day. Then, the cells were analyzed by flow cytometry. Representative flow cytometry graphs and bar charts of 2NBDG uptake (HC, n = 6; D-LC, n = 4) **(A)**, JC-1 (HC, n = 6; D-LC, n = 6) **(B)**, MitoSOX red (HC, n = 11; D-LC, n = 10) **(C)** and MitoTracker green (HC, n = 6; D-LC, n = 7) **(D)** are shown. The data are shown as the median. ns, no statistical significance; *p <0.05.

We further examined B cell glucose uptake after 24 h activation using 2-NBDG, as glucose is an important substrate in B cell activation, and found that 2-NBDG levels in D-LC B cells were significantly lower than those in HC B cells (**Figure 5A**).

Mitochondrial membrane potential (MMP) was also analyzed with JC-1, and the results showed that activated B cells exhibited a reduction in MMP, and the MMPs of B cells from D-LC patients were significantly lower than those from HCs (**Figure 5B**). As a normal MMP is essential for mitochondrial ATP production (19), a decrease in MMP indicates impaired OXPHOS in D-LC B cells, which was in accordance with the reduction in OCR levels (**Figure 4A**).

It has been shown that increased reactive oxygen species (ROS) cause mitochondrial oxidative stress, leading to mitochondrial damage (20). Mitochondrial ROS was measured using MitoSOX and showed that B cells from D-LC patients had increased levels of mitochondrial ROS (**Figure 5C**).

Mitochondrial mass, which is another parameter that represents mitochondrial function, was analyzed, but we did not find any difference between D-LC B cells and HC B cells (**Figure 5D**).

### AKT/mTOR Pathway Changes in B Cells From Decompensated Cirrhosis Patients After T Cell-Dependent Activation

**Figure 6:**
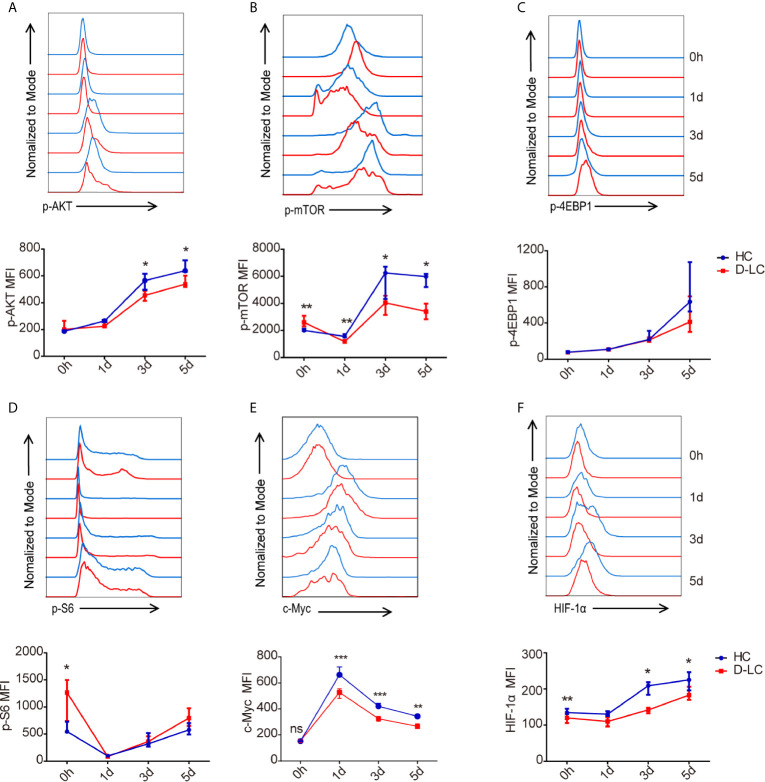
mTOR activity in CD19^+^ B cells was inhibited after T cell-dependent activation in D-LC. CD19^+^ B cells from HCs or D-LC patients were cocultured with cTfh cells. The expression of p-AKT (HC, n = 7; D-LC, n = 6) **(A)**, p-mTOR (HC, n = 7; D-LC, n = 6) **(B)**, p-4EBP1 (HC, n = 6; D-LC, n = 5) **(C)**, p-S6 (HC, n = 6; D-LC, n = 5) **(D)**, c-Myc (HC, n = 7; D-LC, n = 1) **(E)** and HIF-1α (HC, n = 6; D-LC, n = 6) **(F)** was analyzed by flow cytometry. The data are shown as the median (interquartile range). *p <0.05; **p <0.01; ***p < 0.001.

Because the AKT/mTOR pathway is the main signaling pathway that regulates energy metabolism during B cell activation (21), B cells were cocultured with cTfh cells, and the phosphorylation of AKT and mTOR was examined at baseline and on days 1, 3, and 5. In accordance with the previous results, the phosphorylation of AKT and mTOR in D-LC B cells was also impaired in comparison with that in HC B cells (**Figures 6A, B** **)**. Interestingly, the phosphorylation of S6 and 4EBP1 in B cells, which are classic downstream molecules of the AKT/mTOR signaling pathway (22), showed no difference between HCs and D-LC patients (**Figures 6C, D** **)**. Moreover, the levels of c-Myc and HIF-1α, which are downstream molecules of mTOR, were lower in D-LC B cells than in HC cells (**Figures 6E, F** **)**.

### Pretreatment of B Cells With mTOR Pathway Inhibitors Could Inhibit B Cell Differentiation and Proliferation After T Cell-Dependent Activation

**Figure 7:**
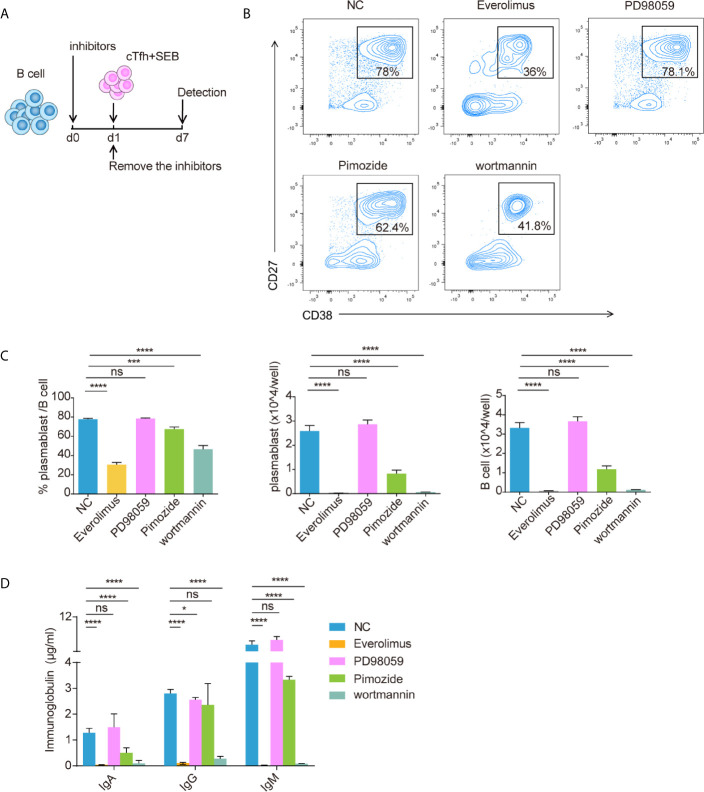
Blocking the mTOR pathway inhibited CD19^+^ B cell differentiation. Isolated healthy CD19^+^ B cells were pretreated with everolimus (1 μM), PD98059 (10 μM), pimozide (7.5 μM) or wortmannin (10 μM) for one day and then cocultured with cTfh cells after the inhibitors were removed **(A)**. Representative flow cytometry graphs **(B)** and bar charts **(C)** show the percentage and number of plasmablasts (CD38^+^CD27^+^) among CD19^+^ B cells. **(D)** The levels of IgA, IgG and IgM in coculture supernatants. The data are shown as the median. *p <0.05; ***p <0.001; and ****p <0.0001.

To verify the effect of the mTOR pathway on T cell-dependent activation, B cells were pretreated with different small molecular inhibitors for one day, after which the inhibitors were removed, and cTfh cells and SEB were added and incubated for another 6 d (**Figure 7A**). The inhibitors used were the mTOR inhibitor everolimus, the MEK inhibitor PD98059, the STAT5 inhibitor pimozide and the PI3K inhibitor wortmannin. The results showed that B cells that were pretreated with everolimus or wortmannin had the lowest percentages and numbers of plasmablasts (CD38^+^CD27^+^) and the lowest IgA, IgG, and IgM production (**Figures 7B–D**). According to the extent of inhibition (**Figure 7C**), the numbers of B cells and plasmablasts were almost zero, while the ratio of plasmablasts was 1/2–1/3 that of the negative control. Inhibition of the mTOR pathway may affect B cell survival and proliferation. The STAT5 inhibitor pimozide partially inhibited B cell proliferation and differentiation after T cell-dependent activation (**Figures 7B–D**).

## Discussion

In this study, we provided an extensive description of the phenotypes, proliferation ability and differentiation capacity of B cells from patients with HBV D-LC after T cell-dependent activation. The proliferation and differentiation capacities of D-LC B cells was impaired after T cell-dependent activation. Mechanistically, we found that B cells from D-LC patients had reduced OXPHOS and glycolysis after activation, which may result from reduced glucose uptake, mitochondrial dysfunction (reduced MMP and increased mitochondrial ROS), and attenuated activation of the AKT/mTOR pathway. Reductions in energy metabolism play a critical role in B cell hyporesponsiveness upon antigen-specific stimulation among patients with HBV D-LC.

B cells in HBV-related advanced cirrhosis had a lower percentage of memory B cells, a higher ratio of plasmablasts and an increased percentage of naïve B cells, which was in accordance with the findings of other studies (10, 11). Memory B cells were chronically activated in advanced cirrhosis, as memory B cells in advanced cirrhosis were more easily activated by TLR9 plus elevated PAMP levels in cirrhotic conditions (10, 11). Activated memory B cells differentiate into short-lived antibody-secreting plasmablasts, increasing the percentage of plasmablasts and the consumption of memory B cells (23, 24). The ratio of naïve B cells was therefore also increased. With regard to memory B cell subgroups, TLMs were thought to be an exhausted phenotype, with increased expression of the inhibitory receptor Fc receptor-like protein 4 (FCRL4) and impaired proliferation (25, 26). The skew toward TLMs in memory B cells in HBV-related advanced cirrhosis in our study suggested an attenuated immune response. Apart from these cells, we also found that the percentage of marginal zone B cells in D-LC patients was decreased. MZB cells could be activated by polysaccharide from encapsulated bacteria independent of T cells (27, 28) The decrease in MZB cells may partially contribute to the susceptibility of decompensated patients to pathogens. The cause of the decrease in MZB cells was not clear. The increased level of bacterial polysaccharides originating from the leaky gut in advanced cirrhosis may lead to persistent MZB cell activation and differentiation, reducing the cell number (4).

Peripheral B cells from both D-LC patients and HCs were cocultured with cTfh cells and showed that B cells from D-LC patients had impaired proliferation and differentiation abilities. Isolated naïve B cells and memory B cells were cocultured with cTfh cells, and the results were consistent with the total B cells, which suggested that the proliferation and differentiation abilities of both naïve and memory subsets were severely influenced. Therefore, the total B cells from D-LC patients showed impaired proliferation and differentiation abilities after activation, not only owing to the lower percentage of memory B cells in D-LC, which was more quickly and robustly to proliferation and differentiation than naïve B cells after activation, but also owing to metabolic abnormal.

Metabolic processes shape immune cell function. Glycolysis is important in IL-4-mediated B cell survival (29). Metabolic reprogramming after B cell activation is important in cell survival, proliferation, differentiation and immunoglobin secretion (12, 29–31). Our findings indicated that B cells from D-LC patients exhibited reduced levels of OXPHOS and glycolysis after IgM activation. Generally, in activated cells, glycolysis has received great attention, as glycolysis is highly increased, and metabolites originating from glycolysis compensate for the need for biosynthesis (30, 32). However, OXPHOS provides 85% of the total ATP in activated PBMCs (33), which suggests that OXPHOS is also inevitable. In addition, B cells were shown to proportionally increase OXPHOS and glycolysis after activation compared with that of T cells (12). This finding indicated that OXPHOS also played an important role at the onset of B cell activation. Regarding the metabolic phenotype, the ratio of OCR/ECAR was reduced in D-LC patients, which indicated that the impairment in OXPHOS was more severe than that in glycolysis, and impaired OXPHOS also led to D-LC B cell dysfunction after activation.

The mTOR pathway has emerged as a critical integrator that receives environmental cues and regulates multiple cellular processes (34). Among these processes are autophagy, glucose uptake and consumption (glycolysis) and the control of protein and lipid synthesis, all of which are central during immune cell activation (34, 35). The activity of mTOR and its upstream factor AKT was attenuated in D-LC B cells after activation. Unexpectedly, the phosphorylation of 4EBP1 and S6, which are effectors downstream of the mTOR pathway, was not changed in D-LC B cells.

In addition to 4EBP1 and S6, mTOR can influence cell death, the CD4+ T cell immune response and T cell metabolism by regulating c-Myc and HIF-1α (36–38). c-Myc and HIF-1α are two main regulators of T cell and B cell metabolism, especially glycolysis and glucose uptake (12, 39, 40). HIF-1α regulates the expression of enzymes in the glycolytic pathway, as well as the expression of the glucose transporters GLUT1 and GLUT3, which mediate cellular glucose uptake. In addition to glucose metabolism, c-Myc also regulates glutaminolysis, an important metabolic process in lymphocyte activation (40). In the present study, the expression of c-Myc and HIF-1α was decreased in D-LC B cells after activation, which indicated that the metabolic impairment after B cell activation was due to c-Myc and HIF-1α downregulation. This result suggested that upon D-LC B cell activation, mTOR functioned *via* crosstalk with c-Myc and HIF-1α. In summary, impaired AkT/mTOR pathway activity may influence cirrhotic B cell metabolism after activation by inhibiting c-Myc and HIF-1α expression.

There were some limitations in this study. First, the enrolled patients all had advanced HBV-related cirrhosis, and cirrhosis caused by other etiologies still needs to be investigated. Because all cirrhotic patients showed cirrhosis-associated immune dysfunction, as indicated by increased levels of inflammatory cytokines and immunodeficiency regardless of etiology (4), we may infer that B cells from patients with decompensated cirrhosis caused by other etiologies will show the same conditions as those from patients with HBV-related cirrhosis. Second, although we explained the attenuation of cirrhotic B cell function from an energy metabolism perspective, we only examined changes in mitochondrial dysfunction and the expression of the main molecular signals, and the cause and detailed regulatory mechanisms are still unknown. As there are many pathogen-associated molecular patterns (PAMPs) and inflammatory cytokines in advanced cirrhosis that cause dysfunction in many other immune cells (4), we should investigate whether PAMPs or cytokines lead to B cell energy metabolism dysfunction. Third, mitochondrial dysfunction was examined, and the reduced MMP and increased ROS levels contributed to OCR. However, the causality between mitochondrial dysfunction and the mTOR pathway has not been elucidated. Fourth, the clone of anti-CD27 antibody used in naïve B and memory B isolation purity detecting was M-T271, which was not suggested in the isolation purity checking, as this clone was the one which was conjugated to the microbeads to do the isolation procedure. This may be the reason for the lower naïve B and memory B isolation purity when detecting by flow cytometry.

Taken together, these data support the hypothesis that B cells from patients with HBV D-LC show impaired proliferation and differentiation after T cell-dependent activation and that this dysfunction occurs because of attenuated increases in OXPHOS and glycolysis after activation, which originated from mitochondrial dysfunction and reduced activity of the main regulatory molecules. Understanding the regulations of B cell metabolic pathway and their changes may provide a new direction to rescue B cell hyporesponsiveness in patients with HBV D-LC, preventing these patients be infected and improving sensitivity to vaccines.

## Data Availability Statement

The raw data supporting the conclusions of this article will be made available by the authors, without undue reservation.

## Ethics Statement

The studies involving human participants were reviewed and approved by the Ethics Committee of First Affiliated Hospital, Zhejiang University School of Medicine. The patients/participants provided their written informed consent to participate in this study.

## Author Contributions

CH wrote this paper. LX helped revise the manuscript. JS, CL, TG and FW were assigned for sample collection. HG, XZ provided the sample. CH, JS and CL performed experiments. CH participated in analysis of the data. LX and ZC conceived the study and participated in its design and coordination. All authors contributed to the article and approved the submitted version.

## Funding

This study was supported by the grants from the National Science and Technology Major Project of China (2018ZX10302206, and 2017ZX10201021-008-001), National Natural Science Foundation of China (81790634) and Scientific research open fund of the Zhejiang Chinese Medical University (491010-X21603).

## Conflict of Interest

The authors declare that the research was conducted in the absence of any commercial or financial relationships that could be construed as a potential conflict of interest.

## Supplementary Material

Supplementary Figure 1 The purity assessment of objective cells before, during and after isolation by flow cytometry. **(A)** The representative chart of purity assessment of cTfh cells. **(B)** The representative chart of purity assessment of positive selection for CD19^+^ cells. **(C)** The representative chart of purity assessment of naïve B cells and memory B cells.

Supplementary Figure 2 Flow cytometry gating strategy of B cell subsets. **(A)** Lymphocytes were firstly gated on a forward scatter (FSC) and side scatter (SSC) plot and then gated based on FSC-H/FSC-A to exclude doublets. B cells were defined as CD19 positive and then B cells were divided into CD10^-^ and CD10^+^ subpopulations. In CD10^+^ subpopulation, transitional B cells were gated as CD38^+^IgD^+^. In CD10^-^ subpopulation, by CD38 and IgD, three groups could be defined, unswitched B cells (CD38^-^IgD^+^), memory B cells (CD38^-^IgD^-^) and plasmablasts (CD38^high^IgD^-^). The unswitched B cells can further divided into CD27^+^ marginal zone B cells and CD27^-^ naïve B cells. Memory B cells were further divided in four subsets based on their maturation state by expression patterns of CD21 and CD27, active memory B cells (AMs, CD21^-^D27^+^), resting memory B cells (RMs, CD21^+^CD27^+^), tissue-like memory B cells (TLMs, CD21^-^CD27^-^) and intermediate memory B cells (IMs, CD21^+^CD27^-^). **(B, C)** The representative graphs of FMO stain and full stain of CXCR3, CXCR4, CXCR5, CCR7, and IL-21R.

Supplemental Methods Tfh cell isolation. At first, CD4^+^ T cells were negatively sorted from PBMCs which were collected from healthy volunteers, using CD4^+^ T cell isolation Kit (Miltenyi Biotec, cat:130096533). Specifically, PBMCs were firstly incubated with Biotin-Antibody Cocktail (a cocktail of biotin-conjugated monoclonal antibodies against CD8a, CD14, CD15, CD16, CD19, CD36, CD56, CD123, TcRγ/δ, and CD235a), which can be conjugated with all the cells in PBMCs except CD4^+^ T cells. Then, MicroBead Cocktail (microbeads conjugated to monoclonal anti-biotin antibody) was added. After incubation, PBMCs were passed through a magnetic column. Collect the flow-through and the separated unlabeled CD4^+^ T cells was in it. Then the selected CD4^+^ T cells were incubated with PE-labeled anti-CXCR5 antibodies. After removing free antibodies, the CD4^+^ T cells subsequently incubated microbeads conjugated to monoclonal anti-PE antibodies (Miltenyi Biotec, cat: 130048801). The cell suspension finally went through a magnetic column, and the microbeads labeled CXCR5^+^ Tfh cells were stay in the column. Memory B cell and naïve B cell isolation. Memory B cell isolation kit (Miltenyi Biotec, cat: 130093546) was used. First, the non–B cells in PBMCs were labeled with a cocktail of biotin-conjugated antibodies (antibodies against CD2, CD14, CD16, CD36, CD43, and CD235a). Second, anti-biotin monoclonal antibodies conjugated to microbeads were added as a secondary labeling reagent. Third, the labeled cells were depleted by magnetic column and unlabeled CD19^+^ B cells were separated. The separated unlabeled CD19+ B were then directly labeled with microbeads conjugated with anti-CD27 antibodies. The magnetic column was used to separate the labeled CD27+ memory B cells and unlabeled CD27- naïve B cells.

## Abbreviations

ACLF, acute-on-chronic liver failure; AD, acute decompensation; AM, active memory B cell; C-LC, compensated liver cirrhosis; cTfh, circulating T follicular cell; D-LC, decompensated liver cirrhosis; ECAR, Extracellular Acidification Rate; ELISA, enzyme linked immunosorbent assay; GLUT1, glucose transporter 1; HAV, hepatitis A virus; HC, healthy control; IM, intermediate memory B cell; MAIT, mucosal-associated invariant T; MMP, mitochondrial membrane potential; MZB, marginal zone B cell; OCR, Oxygen Consumption Rate; OXPHOS, oxygen phosphorylation; PAMP, pathogen associated molecular patterns; PBMC, peripheral blood mononuclear cell; PRR, pattern recognition receptor; RM, resting memory B cell; ROS, reactive oxygen species; SEB, staphylococcal enterotoxin B; TLM, tissue-like memory B cell.
