# Supplementary figures and images for: Reduced Energy Metabolism Impairs T Cell-Dependent B Cell Responses in Patients With Advanced HBV-Related Cirrhosis
Source: Front Immunol. 2021 Jun 23;12:660312. doi: 10.3389/fimmu.2021.660312 (PMC8261287; doi:10.3389/fimmu.2021.660312)

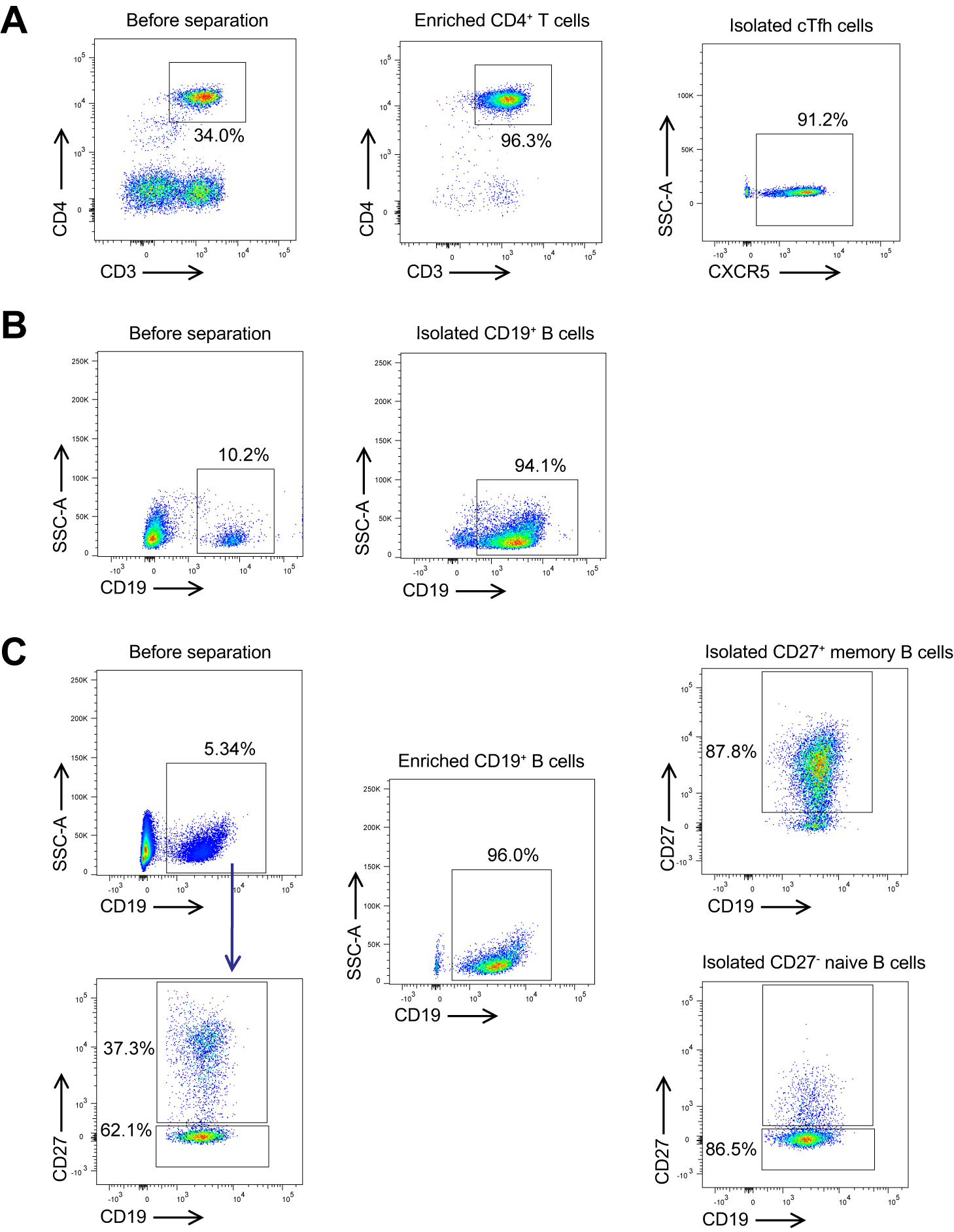

Supplement: Supplementary Figure 1 — The purity assessment of objective cells before, during and after isolation by flow cytometry. (A) The representative chart of purity assessment of cTfh cells. (B) The representative chart of purity assessment of positive selection for CD19+ cells. (C) The representative chart of purity assessment of naïve B cells and memory B cells. [file Image_1.tif]

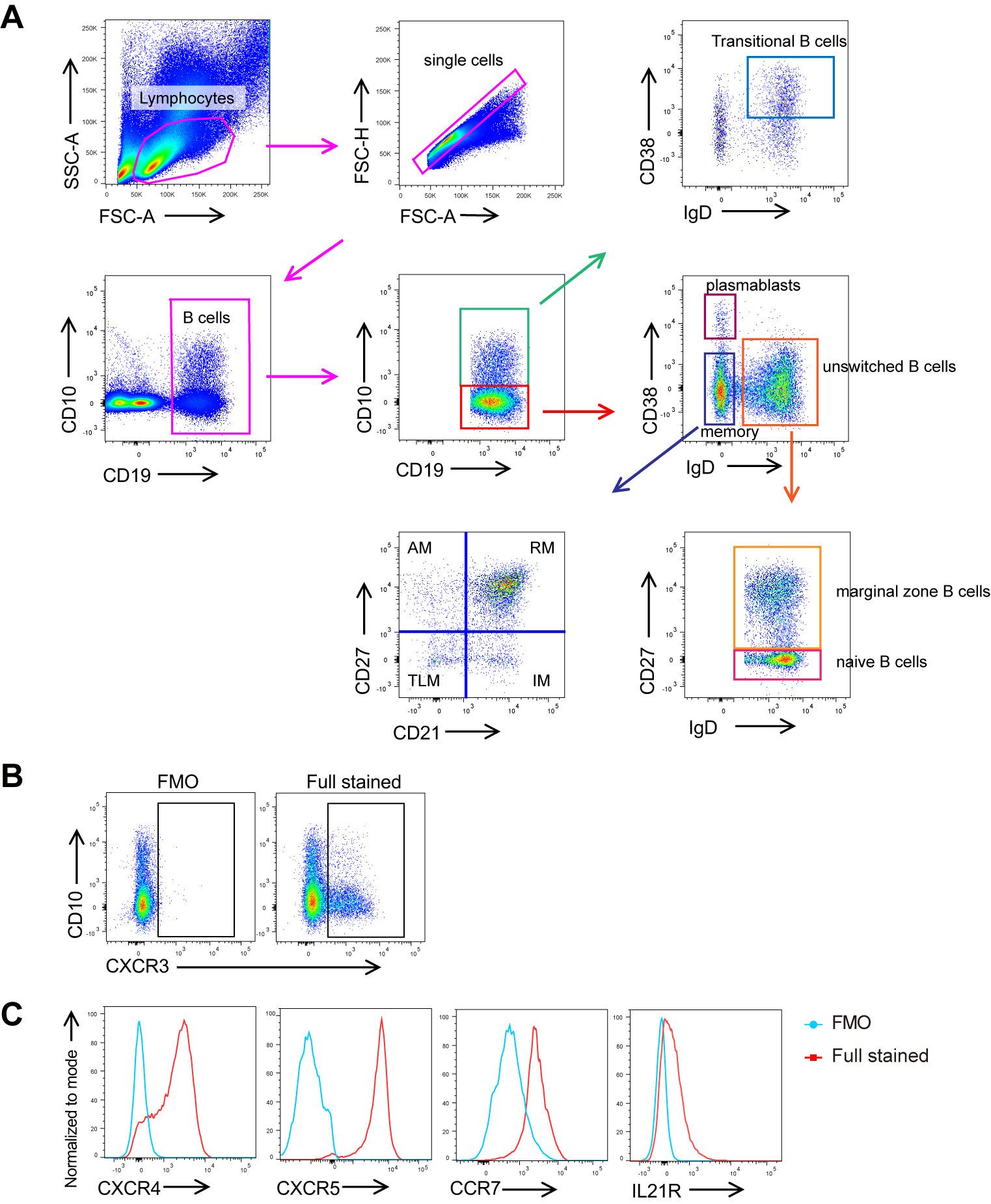

Supplement: Supplementary Figure 2 — Flow cytometry gating strategy of B cell subsets. (A) Lymphocytes were firstly gated on a forward scatter (FSC) and side scatter (SSC) plot and then gated based on FSC-H/FSC-A to exclude doublets. B cells were defined as CD19 positive and then B cells were divided into CD10- and CD10+ subpopulations. In CD10+ subpopulation, transitional B cells were gated as CD38+IgD+. In CD10- subpopulation, by CD38 and IgD, three groups could be defined, unswitched B cells (CD38-IgD+), memory B cells (CD38-IgD-) and plasmablasts (CD38highIgD-). The unswitched B cells can further divided into CD27+ marginal zone B cells and CD27- naïve B cells. Memory B cells were further divided in four subsets based on their maturation state by expression patterns of CD21 and CD27, active memory B cells (AMs, CD21-D27+), resting memory B cells (RMs, CD21+CD27+), tissue-like memory B cells (TLMs, CD21-CD27-) and intermediate memory B cells (IMs, CD21+CD27-). (B, C) The representative graphs of FMO stain and full stain of CXCR3, CXCR4, CXCR5, CCR7, and IL-21R. [file Image_2.tif]
