# Supplementary material for: Reduced Energy Metabolism Impairs T Cell-Dependent B Cell Responses in Patients With Advanced HBV-Related Cirrhosis
Source: Front Immunol. 2021 Jun 23;12:660312. doi: 10.3389/fimmu.2021.660312 (PMC8261287; doi:10.3389/fimmu.2021.660312)
Supplement: Supplemental Methods — Tfh cell isolation. At first, CD4+ T cells were negatively sorted from PBMCs which were collected from healthy volunteers, using CD4+ T cell isolation Kit (Miltenyi Biotec, cat:130096533). Specifically, PBMCs were firstly incubated with Biotin-Antibody Cocktail (a cocktail of biotin-conjugated monoclonal antibodies against CD8a, CD14, CD15, CD16, CD19, CD36, CD56, CD123, TcRγ/δ, and CD235a), which can be conjugated with all the cells in PBMCs except CD4+ T cells. Then, MicroBead Cocktail (microbeads conjugated to monoclonal anti-biotin antibody) was added. After incubation, PBMCs were passed through a magnetic column. Collect the flow-through and the separated unlabeled CD4+ T cells was in it. Then the selected CD4+ T cells were incubated with PE-labeled anti-CXCR5 antibodies. After removing free antibodies, the CD4+ T cells subsequently incubated microbeads conjugated to monoclonal anti-PE antibodies (Miltenyi Biotec, cat: 130048801). The cell suspension finally went through a magnetic column, and the microbeads labeled CXCR5+ Tfh cells were stay in the column. Memory B cell and naïve B cell isolation. Memory B cell isolation kit (Miltenyi Biotec, cat: 130093546) was used. First, the non–B cells in PBMCs were labeled with a cocktail of biotin-conjugated antibodies (antibodies against CD2, CD14, CD16, CD36, CD43, and CD235a). Second, anti-biotin monoclonal antibodies conjugated to microbeads were added as a secondary labeling reagent. Third, the labeled cells were depleted by magnetic column and unlabeled CD19+ B cells were separated. The separated unlabeled CD19+ B were then directly labeled with microbeads conjugated with anti-CD27 antibodies. The magnetic column was used to separate the labeled CD27+ memory B cells and unlabeled CD27- naïve B cells. [file Table_1.doc]

Supplementary Table 1. Antibodies and reagents

| Antibodies and reagents | clone | Manufacturers |
| --- | --- | --- |
| anti-CD4-FITC | RPA-T4 | BD |
| anti-CD3-FITC | OKT3 | Biolegend |
| anti-CD38-PE | HB-7 | Biolegend |
| anti-CD27-APC | M-T271 | BD |
| Fixable Viability Dye eFluor™ 780 |  | ebioscience |
| 7AAD |  | Biolegend |
| DAPI |  | Biolegend |
| anti-CXCR5-PE | J252D4 | Biolegend |
| anti-Ki-67-APC | Ki-67 | Biolegend |
| anti-AKT(pS473)-BV421 | M89-61 | BD |
| anti-mTOR(pS2448)-eF660 | MRRBY | ebioscience |
| anti-HIF1α-AF647 | 546-16 | Biolegend |
| anti-4EBP1 (pT36/pT45)-Alexa 647 | M31-16 | BD |
| anti-S6(Ser235/236)-AF488 | D57.2.2E | CST |
| anti-CD19-FITC | HIB19 | Biolegend |
| anti-CD19-PerCP-Cy5.5 | HIB19 | Biolegend |
| anti-CD10-PE | HI10a | Biolegend |
| anti-CD38-AF700 | HB-7 | Biolegend |
| anti-IgD-BUV395 | IA6-2 | BD |
| anti-CD21-PE/Dazzle™ 594 | Bu32 | Biolegend |
| anti-CD27-BV421 | M-T271 | Biolegend |
| anti-CXCR3-BV510 | G025H7 | Biolegend |
| anti-IL-21R-PE-CY7 | 17A12 | Biolegend |
| anti-CXCR4-BV785 | 12G5 | Biolegend |
| anti-CXCR5-PerCP-Cy5.5 | J252D4 | Biolegend |
| anti-c-Myc-AF594 | 9E10 | Biolegend |
| anti-CCR7-BV605 | G043H7 | Biolegend |
| anti-CD27-PE | O323 | Biolegend |
| Anti-CD3-APC | UCHT1 | BD |
